# Human Leptospirosis Caused by a New, Antigenically Unique *Leptospira* Associated with a *Rattus* Species Reservoir in the Peruvian Amazon

**DOI:** 10.1371/journal.pntd.0000213

**Published:** 2008-04-02

**Authors:** Michael A. Matthias, Jessica N. Ricaldi, Manuel Cespedes, M. Monica Diaz, Renee L. Galloway, Mayuko Saito, Arnold G. Steigerwalt, Kailash P. Patra, Carlos Vidal Ore, Eduardo Gotuzzo, Robert H. Gilman, Paul N. Levett, Joseph M. Vinetz

**Affiliations:** 1 Division of Infectious Diseases, Department of Medicine, University of California San Diego School of Medicine, La Jolla, California, United States of America; 2 Alexander von Humboldt Institute of Tropical Medicine, Universidad Peruana Cayetano Heredia, Lima, Peru; 3 Leptospirosis Reference Laboratory, National Institute of Health, Lima, Peru; 4 CONICET (Consejo de Investigaciones Científicas y Técnicas) and PIDBA (Programa de Investigaciones de Biodiversidad Argentina), Universidad Nacional de Tucumán, Tucumán, Argentina; 5 Leptospirosis Laboratory, Meningitis and Special Pathogens Branch, Centers for Disease Control and Prevention, Atlanta, Georgia, United States of America; 6 Asociacion Benefica PRISMA, Lima, Peru; 7 Directorate of Public Health, Ministry of Health, Loreto Department, Iquitos, Peru; 8 Department of International Health, Johns Hopkins Bloomberg School of Public Health, Baltimore, Maryland, United States of America; 9 Saskatchewan Disease Control Laboratory, Regina, Saskatchewan, Canada; Institut Pasteur, France

## Abstract

As part of a prospective study of leptospirosis and biodiversity of *Leptospira* in the Peruvian Amazon, a new *Leptospira* species was isolated from humans with acute febrile illness. Field trapping identified this leptospire in peridomestic rats (*Rattus norvegicus*, six isolates; *R. rattus*, two isolates) obtained in urban, peri-urban, and rural areas of the Iquitos region. Novelty of this species was proven by serological typing, 16S ribosomal RNA gene sequencing, pulsed-field gel electrophoresis, and DNA-DNA hybridization analysis. We have named this species “*Leptospira licerasiae*” serovar Varillal, and have determined that it is phylogenetically related to, but genetically distinct from, other intermediate *Leptospira* such as *L. fainei* and *L. inadai*. The type strain is serovar Varillal strain VAR 010^T^, which has been deposited into internationally accessible culture collections. By microscopic agglutination test, “*Leptospira licerasiae*” serovar Varillal was antigenically distinct from all known serogroups of *Leptospira* except for low level cross-reaction with rabbit anti–*L. fainei* serovar Hurstbridge at a titer of 1∶100. *LipL32*, although not detectable by PCR, was detectable in “*Leptospira licerasiae*” serovar Varillal by both Southern blot hybridization and Western immunoblot, although on immunoblot, the predicted protein was significantly smaller (27 kDa) than that of *L. interrogans* and *L. kirschneri* (32 kDa). Isolation was rare from humans (2/45 *Leptospira* isolates from 881 febrile patients sampled), but high titers of MAT antibodies against “*Leptospira licerasiae*” serovar Varillal were common (30%) among patients fulfilling serological criteria for acute leptospirosis in the Iquitos region, and uncommon (7%) elsewhere in Peru. This new leptospiral species reflects Amazonian biodiversity and has evolved to become an important cause of leptospirosis in the Peruvian Amazon.

## Author Summary

Leptospirosis has emerged as a globally important infectious disease. Its impact on public health is often difficult to determine, sometimes because of low clinical suspicion, or, as is more common, difficulty in laboratory diagnosis. Gold-standard serology-based diagnosis has a number of important limitations, including the need to use live leptospires that have a sufficient diversity of antigens to be able to detect specific anti-leptospiral antibodies; such antigens vary greatly from region to region. In this paper, we report the discovery of a new species of *Leptospira* in the highly biodiverse region of the Peruvian Amazon, and demonstrate that the animal source of infection for humans is the domestic rat. Detailed biological characterization of this new species shows that it is antigenically unique and represents a new serogroup and serovar, proposed as *Leptospira licerasiae* serogroup Iquitos serovar Varillal. Incorporation of this new isolate into serological testing of patients presenting with acute febrile illness in Iquitos, Peru, showed a far higher incidence of leptospirosis than previously suspected, showing the important of using region-specific *Leptospira* in diagnosis. The field-to-laboratory approach presented here has general application to the discovery of other emerging pathogens and their impact on human health.

## Introduction

Leptospirosis is a zoonotic disease of world-wide distribution caused by pathogenic spirochetes of the genus *Leptospira* [1]–[3]. The disease cannot be diagnosed on clinical grounds alone because its clinical presentations are diverse, ranging from undifferentiated fever to fulminant disease typified by various combinations of jaundice, renal failure, hemorrhage, and shock as well as involvement of other organs such as gallbladder, pancreas, myocardium, and central nervous system. The diagnosis of leptospirosis is made even more difficult by the lack of sensitive and readily accessible diagnostics.

With its diverse fauna, tropical climate and the lack of proper sanitation, the Peruvian Amazon region of Iquitos and its surrounding areas provide an ideal ecological setting for the maintenance and transmission of leptospirosis [1]–[3]. Clinical leptospirosis has neither been commonly recognized nor reported in Iquitos, so that it has been mostly ignored as a cause of febrile illness. In the Iquitos region, as is the case in developing countries around the world, many patients with undifferentiated febrile illnesses do not have an etiology identified, even in comprehensive, prospective studies [4]. Malaria and dengue are important causes of acute febrile illness in the Iquitos region but leptospirosis has only been reported there when research studies have specifically looked for it [5]. Renal carriage among wild animals in Iquitos is common [6], yet comparatively few strains have been isolated in the Peruvian Amazon region of Iquitos [7]. In the context of a prospective study to determine the proportion of acute, differentiated febrile illnesses caused by acute leptospirosis, we isolated a new leptospiral species and serovar. We have provisionally named this isolate “*Leptospira licerasiae”* serovar Varillal strain VAR 010^T^, determined its major mammalian reservoir, and shown its importance in regional diagnosis of acute leptospirosis.

## Materials and Methods

### Humans: Enrollment, Sampling and Culture

Patients presenting at the Belen, Moralillo, Varillal, Padrecocha and Zungarococha Ministry of Health health posts and the Hospital de Apoyo in the Iquitos region of the Peruvian Amazon with complaint of fever were enrolled in a prospective study after oral assent for adults (after reading a detailed script of what participation would consist of along with potential risks and benefits) or written informed consent from parents or legal guardians for children. Included in the informed consent process was a request to administer a questionnaire that asked for personal, medical, demographic and economic information, and requests for serial samples of blood and urine. Specific dates of the study periods are as follows: Belen, January 2003 to September 2005; Hospital Apoyo de Iquitos, May 2003–April 2006; Zungarococha, November 2002 to July 2005; Moralillo, January 2003 to January 2005; Varillal, November 2002 to July 2005; Padre Cocha February 2004 to May 2005.

Inclusion criteria were a self-reported undifferentiated febrile illness of ≤2 weeks duration with a negative malaria smear. Clinical and demographic data were collected from the patients using a questionnaire. Seven milliliters of whole blood were collected by venipuncture at the time of presentation for culture and serological analysis. Follow-up blood samples for serological analysis and mid-stream urine samples for leptospiral culture were collected 10–70 days after enrollment. For urine culture, the pH of samples was adjusted to ∼7.4 with 10 M NaOH at the time of collection. Two tubes of 5 ml semisolid EMJH (Difco, BD Biosciences, Sparks, MD) containing 0.01% (w/v) 5-fluorouracil (5-FU) and 300 µg/ml neomycin were inoculated on site with 2 and 4 drops of whole blood, respectively, using strict aseptic techniques. Urine samples were centrifuged briefly at ∼800 rpm and 2 tubes of semisolid EMJH medium (BD Biosciences) containing the same antibiotics and concentrations were inoculated with 2 and 4 drops of clarified urine, respectively. Cultures were examined weekly by darkfield microscopy and classified as negative if no organisms typical of *Leptospira* were observed by 12 weeks. A high level of care was taken to avoid contamination by water-borne saprophytic *Leptospira*; no saprophytes were obtained during the course of the study (as determined by 16S rRNA gene sequencing).

This study was approved by the Human Subjects Protection Program, University of California San Diego, and the Ethical Committees of Asociacion Benefica PRISMA, Lima, Peru, and Universidad Peruana Cayetano Heredia, Lima, Peru.

### Human Serological Analysis and Interpretation Criteria

Serologic testing of patient samples was performed at the Instituto Nacional de Salud in Lima, Peru using an in-house IgM ELISA [8] (which includes as antigens serovars Icterohaemorrhagiae, Bratislava, Ballum, Canicola, Cynopteri, Grippotyphosa but not *“L. licerasiae”* serovar Varillal). Microscopic agglutination testing (MAT) was done using the following antigens (serogroup followed by serovar in parentheses): serogroup Andamana (serovar Andamana), Australis (Australis and Bratislava), Ballum (Ballum), Bataviae (Bataviae), Canicola (Canicola), Celledoni (Celledoni), Cynopteri (Cynopteri), Djasiman (Djasiman), Grippotyphosa (Grippotyphosa), Hebdomadis (Borincana), Icterohaemorrhagiae (Copenhageni, Icterohaemorrhagiae and Mankarso), Javanica (Javanica), Mini (Georgia), Pomona (Pomona), Pyrogenes (Alexi and Pyrogenes), Sejroe (Hardjo and Wolffi), and Tarassovi (Tarassovi). Sera were screened at a dilution of 1∶100 and positive sera were titrated to endpoint using standard methods [9].

Clinical criteria for submitting sera on patients (both in Iquitos and nationwide) for serological diagnosis were undifferentiated fever for 2 weeks or less, malaria smear negative, and no alternative explanation for fever.

Serological criteria for diagnosing acute leptospirosis in all areas of Peru other than Iquitos included any one of the following: seroconversion in IgM by ELISA from acute to convalescent sera; seroconversion in MAT from negative to 1∶100 or greater; 4-fold rise in titer between acute and convalescent sera; or a single titer of 1∶400 or greater. The single titer of 1∶400 in non-Iquitos regions was chosen as the seropositivity cutoff because of national data indicating that titers at this level or lower were background in the population in asymptomatic individuals (M. Cespedes, Instituto Nacional de Salud, Lima, Peru, unpublished observations).

Serological criteria for stating that a specific MAT titer was associated with acute leptospirosis were made more stringent in Iquitos than in other parts of Peru because of the high prevalence of low level (1∶400 or less) anti-*“L. licerasiae”* serovar Varillal antibodies in asymptomatic individuals (data not shown). Serological criteria to assign a diagnosis of acute leptospirosis in Iquitos included any one of the following: IgM positive by ELISA in either acute or convalescent sera; seroconversion in MAT from negative to 1∶100 or greater; 4-fold rise in titer between acute and convalescent sera; or a single titer of 1∶800 or greater.

### Animals: Trapping and Culture for *Leptospira*

Rats were caught live in baited wire-mesh traps (Tomahawk, USA) left overnight near dwellings in the urban area of San Juan near the Iquitos airport, in the urban slum of Belen in Iquitos, or the rural area of Moralillo 15 km outside Iquitos removed 1 km from the Iquitos-Nauta road. Dates which animals were captured, according to isolate, are as follows: CEH001, 11/22/02; CEH006, 11/26/02; CEH010 11/26/02; CEH011, 12/14/02; CEH033 12/17/02; CEH044 12/19/02; CEH162 01/21/03; MMD735, 01/19/03. Animals were anesthetized with isoflurane and the kidneys were removed aseptically; blood was collected by cardiac puncture. Excised kidney material was minced using a sterile scalpel blade and cultured in semisolid EMJH containing antibiotics. All cultures were incubated at 30°C for up to 12 weeks and checked bi-weekly for growth. Positive cultures were sub-cultured into liquid EMJH for serological and molecular typing. Animal trapping and use was approved by the Instituto Nacional de Recursos Naturales of Peru (INRENA) and the Institutional Animal Care and Use Committee, University of California San Diego.

### Pulsed Field Gel Electrophoresis (PFGE) Characterization of Isolates

Agarose blocks containing leptospiral DNA were prepared and then digested with 30 units of NotI restriction enzyme (New England Biolabs, USA) for 2 hours at 37°C. Plug slices containing the digested DNA were placed in the wells of a 1% agarose gel and electrophoresed in a Bio-Rad CHEF-DRIII for 18 hours at 14°C with recirculating TBE buffer. Initial and final switch times of 2.16 and 35.07 s, respectively, were employed, and voltage was 6 V/cm. *Salmonella* serotype Braenderup H9812 was digested with 50 U XbaI (New England Biolabs) for use as a DNA size standard [10]. Gels were stained with ethidium bromide and then photographed under UV trans-illumination using the Gel Doc 2000 system (Bio-Rad). PFGE fingerprints were analyzed using the BioNumerics software package (Applied Maths, Belgium) and a database of PFGE profiles from reference strains and clinical isolates (Galloway and Levett, unpublished data). The Dice band-based coefficient was used for cluster analysis [11].

### Characterization of isolates by 16S rRNA Gene Sequencing

Total genomic DNA was extracted from 7 day cultures (2×10^8^ leptospires/mL) using the QIAamp DNA extraction kit (QIAGEN, USA). Initial PCR amplification was performed using the eubacterial rDNA primers fD1/rD1 as described previously for leptospiral 16S rRNA gene sequencing [12]. PCR products were purified using the Qiaquick PCR purification kit (QIAGEN, USA). Sequencing was performed on an ABI 3100 automated sequencer (Perkin Elmer, USA). Since the most informative 16S sequence is found in the middle of the leptospiral 16S rRNA gene, base pairs from ∼32 to 1355 were sequenced, using the following internal sequencing primers: lepto16S11f, a 20 bp forward primer located at position 11 (5′- GGC GGC GCG TCT TAA ACA TGC - 3′); and lepto16S1388r, a 20 bp reverse primer located at position 1388, (5′-TGT GTA CAA GGT CCG GGA AC - 3′). Additional internal sequencing was done using specific forward primers beginning at position 505 (5′- TCA TTG GGC GTA AAG GGT G – 3′) and position 1006 (5′ - TCA GCT CGT GTC GTG AGA TG – 3′). For clarity, the sequencing strategy is available online (Figure S1, a schematic of the PCR and sequencing primer locations and Figure S2, an example of one such sequence assembly). Reads of at least 650–700 bp were routinely obtained. 16S rRNA gene segments were sequenced 8 times in both directions. Given that informative sequence cannot include the PCR primers, ∼1355 bp of informative primary sequence was obtained for each isolate. Reaction conditions for cycle sequencing were according to manufacturer's directions. Sequences were edited and assembled using the Staden Software Package [13]. Edited sequences were aligned using ClustalW v. 1.83 [14] for Mac and a phylogram generated using MrBayes v3.1.2 [15] for Mac with 2 simultaneous runs for 3,000,000 generations. The Tamura-Nei (1993) (TrN+I+G) model of nucleotide substitution with gamma distributed rates and invariant sites was used [16].

### DNA-DNA Hybridization Analysis

Subcultures in liquid PLM-5 medium were incubated at 30°C for 7 days. DNA was extracted and purified from strains VAR010^T^, CEH010, CEH011, CEH033, CEH044, CEH162, MMD735, *L. interrogans* RGA^T^, *L. broomii* 5399^T^, *L. fainei* BUT6^T^ and *L. inadai* Lyme^T^ as described previously [17]. DNA from strain VAR 010^T^ was labeled with [^32^P]dCTP [17] and DNA relatedness and percentage divergence between the strains were determined by the hydroxyapatite method, with 55°C used for optimal reassociation (Table 1).

**Table 1:** DNA relatedness of “*Leptospira licerasiae*” strain VAR010^T^ to other *Leptospira* species: *L. broomii* 5399^T^, *L. fainei* BUT6^T^, *L. inadai* Lyme^T^ and *L. interrogans* RGA^T^.

Source of unlabelled DNA	Results of reaction with labeled DNA from strain VAR010^T^	
	RBR^a^	D
VAR010^T^	100	0.0
CEH010	95	0.7
CEH011	92	0.6
CEH033	98	0.0
CEH044	91	0.4
CEH162	100	0.6
MMD735	96	0.6
*L. broomii* 5399^T^	15	
*L. fainei* BUT6^T^	13	
*L. inadai* Lyme^T^	37	
*L. interrogans* RGA^T^	34	

a RBR, relative binding ratio; D, percent divergence. Reactions were performed at 55°C.

The G+C content (mol%) was determined for strain VAR 010^T^ by the thermal denaturation method using a Beckman DU Series spectrophotometer (Beckman Coulter, Fullerton, CA) [18]. All samples were run at least three times, using DNA from *Escherichia coli* K-12 as a control.

#### Determination of leptospiral serogroup

Leptospiral isolates at a density of 2×10^8^ cells/mL were used in microscopic agglutination reactions with reference rabbit anti-sera raised against the panel of all leptospiral serogroups except for Lyme and Sehgali shown in Table 2 [9]. Individual titers higher than 1∶100 were considered significant and reported.

**Table 2:** Panel of Leptospiral Serogroup Antisera Used to Characterize “*Leptospira licerasiae*” strain VAR10^T^.

Serogroup	Serovar	Strain
Australis	Australis	Ballico
	Bratislava	Jež Bratislava
	Peruviana	941
Autumnalis	Autumnalis	Akiyami A
Ballum	Ballum	Mus 127
Bataviae	Bataviae	Van Tienen
	Rioja	MR 12
Canicola	Canicola	Hond Utrecht IV
Celledoni	Celledoni	Celledoni
Cynopteri	Cynopteri	3522 C
	Tingomaria	M-13
Djasiman	Djasiman	Djasiman
Grippotyphosa	Grippotyphosa	Moskva V
Hebdomadis	Borincana	HS 622
Hurstbridge	Hurstbridge	BUT 6
Icterohaemorrhagiae	Copenhageni	M 20
	Icterohaemorrhagiae	RGA
	Lai	Lai
	Mankarso	Mankarso
Javanica	Javanica	Veldrat Batavia 46
	Vargonicas	24
Louisiana	Orleans	LSU 2580
Manhao	Manhao 4	Li 130
Mini	Georgia	LT 117
	Ruparupae	M 3
Panama	Panama	CZ 214
Pomona	Pomona	Pomona
Pyrogenes	Bagua	MW 12
	Cenepa	MW 2
	Pyrogenes	Salinem
Ranarum	Evansi	267-1348
Sarmin	Machiguenga	MMD 3
	Sarmin	Sarmin
Sejroe	Wolffi	3705
Shermani	Babudieri	CI 40
Tarassovi	Tarassovi	Perepelitsin
Semaranga	Patoc	Patoc 1

### Biological Characterization

#### Growth Characteristics

Growth of the unknown leptospiral isolate was determined in the presence of 225 µg/mL 8-azaguanine (8-AZA) at 30°C [19]. *L. interrogans* serovar Icterohaemorrhagiae strain HAI188 [5], *L. fainei* serovar Hurstbridge strain BUT6^T^, and *L. biflexa* serovar Patoc strain Patoc I^T^ were included as representative pathogenic, intermediate and saprophytic strains, respectively. Growth in liquid EMJH, without 8-AZA, was used as a positive control.

#### *LipL32* PCR

To assess the presence of a PCR-amplifiable *LipL32* gene in *“L. licerasiae”*, we used a modified PCR procedure [20], and used DNA from *L. interrogans* serovars Copenhageni strain L1-130 [21] and HAI188 [5], respectively, as positive controls. All amplifications were performed on the PTC-200 system (MJ Research, Bio-Rad, Hercules, CA). Five µL of genomic DNA was amplified using the following protocol: 95°C for 15 s for enzyme activation, followed by 40 cycles of 95°C for 45 s, 55°C for 30 s and 72°C for 60 s. The annealing temperature was decreased by 1°C per cycle for the first 5 cycles.

#### LipL32 Western Blot

Western blot analysis to detect LipL32 in leptospiral strains was performed using 2×10^7^ leptospires/well separated by SDS-PAGE and transferred to a nitrocellulose membrane. The strains studied included “*L. licerasiae*” serovar Varillal strain VAR 010^T^, *L. interrogans* serovar Icterohaemorrhagiae strain HAI188 [5], *L. fainei* serovar Hurstbridge strain BUT6^T^ (ATCC BAA-1109^T^), as well as *L. broomii* ((ATCC BAA-1107^T^ and BAA-1108), *L. weilii* serovar Celledoni (ATCC 43285^T^), *L. wolbachii* serovar Codice (ATCC 43284^T^), *L. biflexa* serovar Patoc (ATCC 23482^T^), and *Turneriella parva* (ATCC BAA-1111^T^) were obtained from the American Type Culture Collection, Virginia, USA. 100 µl of cultures were taken directly from the glycerol stock and centrifuged at 14,000 rpm for 30 minutes. The pellets were washed three times with PBS, suspended in 100 µl of 1× SDS sample buffer and incubated in a boiling water bath for 10 min. Ten µl of the SDS-solubilized whole bacterial cell lysate were loaded onto 4–12% Bis-Tris SDS polyacrylamide gels (Invitrogen, Carlsbad, USA) and transferred to nitrocellulose membrane. The blot was blocked in PBS containing 5% BSA, incubated in anti-LipL32 rabbit polyclonal antiserum (diluted 1∶2000, 2 hr at 21°C; kindly provided by Dr. David Haake, University of California, Los Angeles) followed by 1 hr incubation with 1∶3000 dilution of phosphatase-labeled anti-rabbit IgG (Kierkegaard and Perry Laboratories, Gaithersberg, Maryland), and development with BCIP/NBT (Kierkegaard and Perry Laboratories).

### Experimental challenge infections by *Leptospira*

Outbred female Syrian Golden hamsters were obtained from Charles River Laboratories (Wilmington, MA). Animal experiments were approved by the University of California, San Diego Institutional Animal Care and Use Committee and were performed in Biosafety Level 2 animal facilities approved by the Association for Assessment and Accreditation of Laboratory Animal Care under approved biological safety procedures. *“L. licerasiae”* strain VAR 010^T^ and 2 other isolates from Iquitos, Peru (*L. interrogans* serovar Icterohaemorrhagiae, strains HAI188 and HAI156) that were isolated from leptospirosis patients were used to infect hamsters (N = 2 in each group). Organisms fixed with 10% formalin were counted using a Petroff-Hauser counting chamber using dark-field microscopy. Groups of hamsters were inoculated intraperitoneally with 10^8^ organisms for each *Leptospira* strain; one animal was injected with EMJH leptospiral culture medium alone as a negative control. The animals were observed twice daily for clinical signs of disease (hunching, decrease in oral intake, diarrhea, lethargy). On day 3 following infection, one member of each group was sacrificed and the organs (lung, liver, and kidney) were removed aseptically to determine the bacterial load by real-time quantitative PCR [5]. The remaining animal in each group was sacrificed if moribund, and the organs were harvested and processed similarly. Samples for PCR were stored in 70% ethanol at −80°C until needed.

### DNA Preparation and Real-Time qPCR

Total DNA for qPCR was prepared from three different pieces of weighed tissue samples using the DNeasy tissue kit (QIAGEN, USA) according to the manufacturer's directions. Real-time qPCR was performed using a previously described primer pair and probe [22] labeled with the fluorescent reporter dye FAM (6-carboxyfluorescein) at the 5′ end, and the fluorescent quencher TAMRA (6-carboxytetramethylrhodamine) at the 3′ end. The PCR primers, Lepto F (5′-CCCGCGCCCGCG TCCGATTAG-3′) and Lepto R (5′ TCCATTGTGGCCGRA/GACAC-3′), allow amplification of the region between the positions 171 and 258 of the rrs (16S) gene, with an expected product size of 87 bp. The FAM-TAMRA labeled probe [5′-CTCACCAAGCTCACCAAG GCGACGATCGGTAGC-3′] spans the region from position 205 to 228. Reaction mixes were prepared using the Platinum Quantitative PCR supermix-UDG kit (Invitrogen, Carlsbad, CA, USA) with final primer and probe concentrations of 600 nM and 100 nM, respectively, and 5 µl DNA extract. Reactions were performed in triplicate. Amplification and fluorescent monitoring were performed using a DNA Engine Opticon® 2 thermal cycler (MJ Research) using the following protocol: Incubate 2 min at 50.0°C; incubate 2 min at 95.0°C; incubate 30 s at 94.0°C; incubate 1 min at 50.0°C; plate read; repeat steps 2–5 for 44 more cycles.

To generate a standard curve, 13 mg of uninfected hamster kidney was spiked with 10^8^ leptospires, extracted as described above, and used to prepare a 10-fold dilution series for real-time qPCR. The tissue burden of *Leptospira* for each sample was quantified by interpolating threshold cycle (Ct) values against the standard curve. Samples with a C_t_ value >40 were considered negative.

### LigA Southern Blot

A dioxigenin (DIG)-labeled 508 bp probe for detection of the pathogenic marker *lig*A gene was synthesized by PCR, using the following forward primer, 5′ - CAAAGTTGTATGTCTTGGCCA C 3 - ′ and reverse primer, 5′ - GGAAGACCAAACGATCAG TGG - 3′. DNA from *L. interrogans* serovar Icterohaemorrhagiae strain HAI0188 was used as template. The PCR cycling profile consisted of 40 cycles of 95°C for 30 s; 49°C for 30 s; 72°C for 40 s; and a final extension of 72°C for 7 min. A 16S rRNA gene probe to be used as a control was generated using primers lepto16S1006f, 5′ - TCAGCTCGTCGTGTCGTGAGATG - 3′, designed from aligned leptospiral 16S sequences retrieved from GenBank, and rD1, 5′- AAGGAGGTGATCCAGCC - 3′ [23]. Genomic DNA was extracted from strain HAI188, *“L. licerasiae”* strain VAR 010^T^, *L. fainei* serovar Hurstbridge strain BUT6^T^, and *L. biflexa* serovar Patoc I^T^ using the DNeasy Tissue Kit (Qiagen, USA), and was then digested with *Bam*HI (New England Biolabs, USA) according to manufacturer's directions. Hybridization was carried out at 42°C. The membrane was washed with 2× SSC at room temperature and 0.1× SSC at 42°C. Bands were detected using anti-DIG-alkaline phosphatase Fab fragments (Roche, USA) and CDP-Star chemiluminescence substrate (Roche, USA).

## Results

### Patient Isolates of *Leptospira*

Of 881 patients presenting with a history of undifferentiated fever to a study site, 45 patients' blood cultures yielded leptospires with typical morphology and motility as visualized under darkfield microscopy. Two of these leptospiral isolates from humans, obtained from blood cultures and identified as novel based on results presented below, were studied further. None of the 881 patients had urine cultures positive for this novel leptospire.

### Case Descriptions of Patients with Novel Leptospires

#### Patient VAR10

A 31 year old female food vendor presented at the Varillal health post complaining of 2 days of fever, malaise, chills, headache and dizziness. She denied having gastrointestinal or urinary symptoms. The physical exam was unremarkable. The blood smear was negative for malaria and the patient was sent home with antipyretics. Illness resolved after 5 days without any further treatment or complications.

#### Patient HAI029

A 19 year-old female student/domestic worker presented at the Hospital de Apoyo in Iquitos with a 5-day history of fever, malaise, headache, dizziness, chills, leg pain and weakness, abdominal pain, anorexia, nausea, and vomiting. Her illness spontaneously resolved with no complications.

#### Follow Up

Neither patients VAR10 nor HAI029 received antibiotic treatment. At 5 week follow up, all signs and symptoms of infection had completely resolved in both patients. Blood cultures from both patients were positive for *Leptospira* at 3 and 2 weeks after inoculation, respectively. The characterization of these leptospiral isolates as a new species and unique antigenic type is described below. The isolate from patient VAR10 (strain VAR 010^T^) has been deposited at the American Type Culture Collection as ATCC BAA-1110^T^, at the U.S. National Veterinary Services Laboratory, Ames, Iowa, USA, and at the WHO/FAO/OIE Collaborating Centre for Reference and Research on Leptospirosis, Australia and Western Pacific Region, Brisbane, Australia.

Serological results for patient VAR10 showed negative ELISA IgM serology on both acute and convalescent samples. MAT using the standard live *Leptospira* panel was negative. When the isolate from patient VAR10 was used as MAT antigen, the acute sample was negative but the convalescent sample (taken 42 days after the acute sample) was positive at a titer of 1∶400.

The acute serum sample of patient HAI029 was negative for anti-leptospiral antibodies but was IgM positive by ELISA on a convalescent sample taken 31 days after the acute sample. The acute serum sample of patient HAI029 was negative by MAT by both the standard leptospiral panel plus *“L. licerasiae*” strain VAR 010^T^ but convalescent samples reacted to serogroups Canicola, Icterohaemorrhagiae, Australis, and Sejroe at a titer of 1∶1600, to serogroup Mini at a titer of 1∶3200, and had a titer of 1∶6400 against her own isolate subsequently identified as *“L. licerasiae”* serovar Varillal, and identical to strain VAR 010^T^ as determined by PFGE and 16S rRNA gene sequencing (see below).

### Leptospiral Isolation from Animals

Peri-domestic rats were trapped in the context of a mammalian ecology study of *Leptospira* reservoir hosts in the Peruvian Amazon. Of 100 *Rattus rattus* and *R. norvegicus* trapped, 55 isolates of *Leptospira* were obtained from culture of kidney. Of these 55 isolates, 47 proved to be *L. interrogans* serovar Icterohaemorrhagiae by testing with serovar-specific reference polyclonal antisera; 8 were not agglutinated by a panel of antisera against the standard serogroups and thus were studied further. Six of these non-serologically-typeable isolates came from *R. norvegicus,* two from *R. rattus.* Of more than 100 isolates obtained from cattle, pigs and water buffalo in the Iquitos region, none had any genetic relatedness to the novel leptospire (data not shown).

### Characterization of Isolates

**Figure 1:**
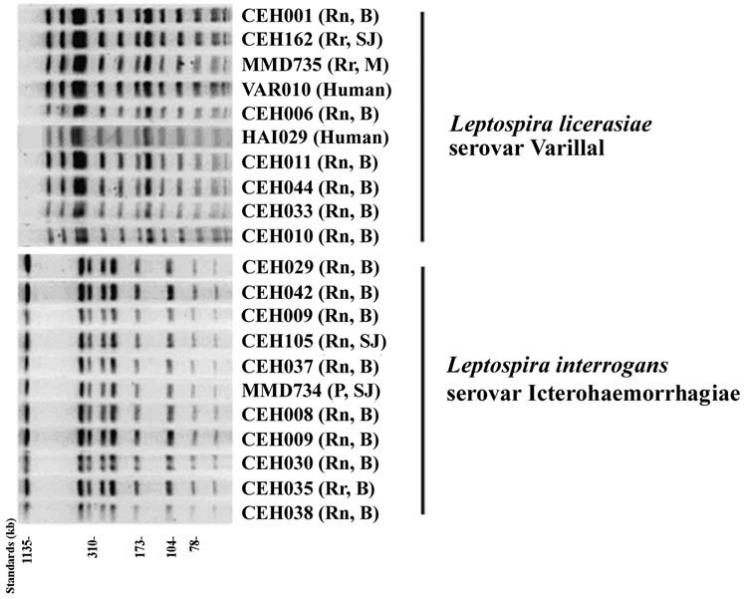
Pulsed field gel electrophoresis analysis of leptospiral isolates obtained from rats and humans in the region of Iquitos, Peru. Indicated in parentheses is animal source of leptospiral isolate followed by location of trapping (see Methods). Rn, *Rattus norvegicus;* Rr, *Rattus rattus*. B, Belen; SJ, San Juan; M, Moralillo.

PFGE analysis was performed on the VAR 010^T^ and HAI029 human isolates and the 8 non-serologically-typeable isolates from rats. PFGE fingerprints were compared to PFGE fingerprints from 206 other pathogenic, intermediate (all those included in this paper) and saprophytic serovars (Galloway and Levett, data not published). These rat and human isolates shared a previously undescribed fingerprint pattern (Figure 1).

### 16S rRNA Gene Sequencing

**Figure 2:**
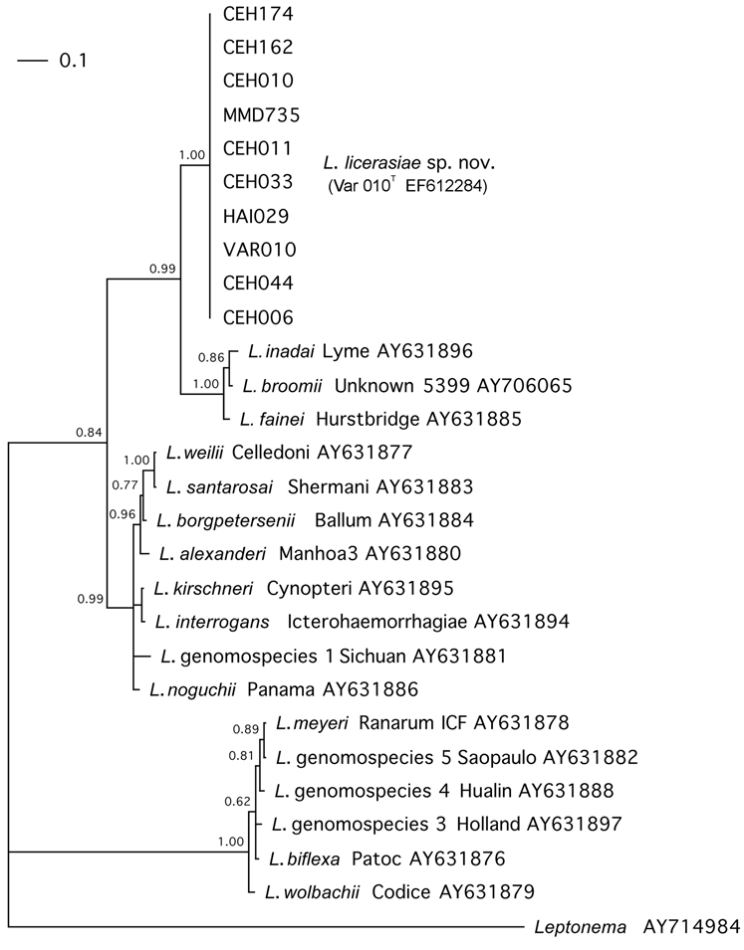
Phylogram of leptospiral 16S rRNA gene sequences generated by Bayesian phylogenetic analysis with simultaneous runs of 3,000,000 generations. Bootstrap confidence in assigning branch points is indicated at each node. For clarity, the intermediate clade of leptospires is placed on top, with the *L. licerasiae* strains first; the intermediates, pathogens and saprophytes groups of *Leptospira* are indicated at the right. *Leptonema* is used as the outgroup for comparison. The scale bar (upper left) shows the fractional difference in 16S rRNA gene nucleotide sequences. GenBank accession numbers are indicated to the right of each strain analyzed.

16S rRNA gene fragments of ∼1.5 kb were amplified from genomic DNA extracted from all isolates in the study using the universal eubacterial primers fD1/rD1 [23]. The internal primers lepto16S505f and lepto16S1006f were designed from consensus regions of published leptospiral 16S rRNA gene sequences and used to sequence an internal ∼1.3 kb portion of the fD1/rD1 fragment. The sequences of the 16S rRNA fragment from all strains with the new PFGE pattern were identical. Phylogenetic analysis revealed that these strains were more closely related to the intermediate leptospiral species *L. fainei* and *L. inadai* (Figure 2) than to the more pathogenic *Leptospira interrogans* group [24].

### Serotyping

The panel of reference rabbit anti-serogroup polyclonal antisera used in this study failed to agglutinate the leptospiral strains isolated from patients VAR10 and HAI029 and the 8 rat isolates in the MAT. *“L. licerasiae”* serovar Varillal strain VAR 010^T^ was agglutinated by antisera to serovar Hurstbridge at a titer of 1∶100 but by no other anti-serogroup antisera. Conversely, no other serogroup was agglutinated by the reference rabbit anti-serum raised against *“L. licerasiae”* serovar Varillal strain VAR 010^T^. Because of the lack of significant seroreactivity of reference serogroup antisera against *“L. licerasiae”* serovar Varillal strain VAR 010^T^, the cross-agglutination absorption test (CAAT) was not carried out. A similar approach was used to designate the Hurstbridge serovar of *L. fainei* serovar [19]. These serotyping results were independently validated at the WHO/FAO/OIE Collaborating Centre for Reference and Research on Leptospirosis, Australia and Western Pacific Region (Dr. Lee Smythe, Table S1). Rabbit anti-serum to *“L. licerasiae”* serovar Varillal strain VAR 010^T^ (available from the National Veterinary Services Laboratory, Ames, Iowa) agglutinated the leptospires from patients VAR10 and HAI029, and the eight rat isolates, at a titer of 1∶51,200. These serological results conclusively demonstrate that the two human and eight rat leptospires represent a new serogroup and a new serovar.

### DNA-DNA Hybridization

Because leptospiral strains VAR 010^T^, CEH010, CEH011, CEH033, CEH044, and CEH162 grouped with the intermediate leptospires by 16S rRNA phylogenetic analysis, DNA-DNA hybridization was only carried out on the other intermediates *L. broomii* 5399^T^, *L. fainei* BUT6^T^ and *L. inadai* Lyme^T^ as well as *L. interrogans* RGA^T^ as an outgroup. As shown by DNA-DNA hybridization analysis (Table 1), leptospiral strains VAR 010^T^, CEH010, CEH011, CEH033, CEH044, and CEH162 showed no significant relatedness to *L. interrogans* RGA^T^, *L. broomii* 5399^T^, *L. fainei* BUT6^T^ or *L. inadai* Lyme^T^. However, there was strong relatedness between the strains VAR 010^T^, CEH010, CEH011, CEH033, CEH044, CEH162 and MMD735. These strains meet the criteria for the molecular definition of a species [25]. The G+C content of *L. licerasiae* strain VAR 010^T^ was 43.9 mol%, within the range reported for other *Leptospira* species [26].

### Biological characterization

To determine whether *“L. licerasiae”* serovar Varillal strain VAR 010^T^ had growth characteristics more typical of pathogenic or saprophytic *Leptospira*, growth in the presence of 8-azaguanine, a classic test to differentiate pathogenic from saprophytic leptospires [19], was performed. *“L. licerasiae”* serovar Varillal strain VAR 010^T^, *L. interrogans* serovar Icterohaemorrhagiae strain HAI188, and *L. fainei* serovar Hurstbridge strain BUT6^T^ failed to grow in the presence of 8-AZA after 4 weeks incubation at 30°C; as a positive control, the saprophytic strain, *L. biflexa* strain Patoc I^T^, grew well in the presence of 8-azaguanine.

**Figure 3:**
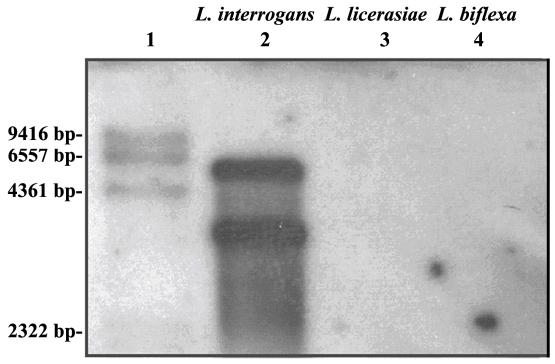
Southern blot to determine the presence of *LigA* in *“L. licerasiae”* serovar Varillal. Lane 1, DIG-labeled marker; lane 2, *L. interrogans* serovar Icterohaemorrhagiae strain HAI188 (positive control); lane 3, *“L. licerasiae”* serovar Varillal; lane 4, *L. biflexa* serovar Patoc I^T^.

Southern blotting and PCR were performed to determine whether the *LigA* gene, encoding the putative virulence factor Lig A found in *L. interrogans*, might be present in *“L. licerasiae”* serovar Varillal strain VAR 010^T^. PCR using 4 pairs of primers derived from *L. interrogans* serovar Copenhageni failed to produce a *LigA* band in *“L. licerasiae”* serovar Varillal strain VAR 010^T^. Southern blot analysis for *Lig A* showed the expected bands in a strain of *L. interrogans* serovar Icterohaemorrhagiae strain HAI188, as expected, but failed to detect *Lig A* in *“L. licerasiae”* serovar Varillal strain VAR 010^T^ or in *L. fainei* serovar Hurstbridge strain BUT6^T^ and *L. biflexa* serovar Patoc strain Patoc I^T^ (Figure 3).

### Demonstration of a LipL32-related protein in “*L. licerasiae*”, *L. fainei* and *L. biflexa*

The presence of the lipoprotein *LipL32* gene has been considered characteristic of pathogenic leptospires [27]. PCR, using published primers [20],[27] to amplify *LipL32*, detected the expected product only in a pathogenic *L. interrogans* serovar Icterohaemorrhagiae strain HAI188, but not in *“L. licerasiae”* serovar Varillal strain VAR 010^T^, HAI029, the rat-derived *“L. licerasiae”* strains, *L. fainei* or *L. biflexa*. However, Southern blotting revealed that in addition to strain HAI188, both *L. fainei* serovar Hurstbridge strain BUT6^T^ and *“L. licerasiae”* serovar Varillal strain VAR 010^T^, but not *L. biflexa,* had bands that hybridized to the *L. kirschneri*-derived *LipL32* probe (data not shown).

**Figure 4:**
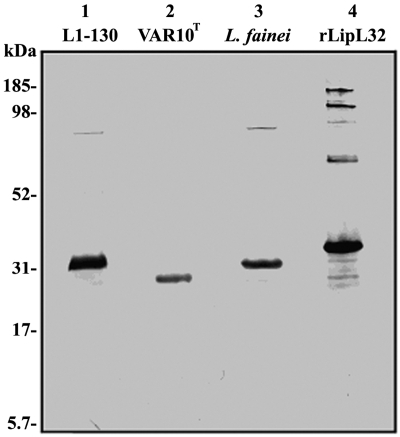
Western immunoblot of *Leptospira interrogans* serovar Copenhageni strain L1-130, *“L. licerasiae”* serovar Varillal, and *L. fainei* serovar Hurstbridge using rabbit polyclonal antisera to recombinant LipL32 of *L. kirschneri* serovar Grippotyphosa. rLipL32, recombinant LipL32 of *L. kirschneri* serovar Grippotyphosa produced in *E. coli*.

Because of the potential for the phylogenetically distant *Leptospira* to have sufficiently diverged so that PCR amplification may have failed because of primer mismatch, we determined whether a LipL32 cross-reactive protein might be present in *“L. licerasiae”* serovar Varillal strain VAR 010^T^ by Western immunoblot using a rabbit anti-*L. kirschneri* serovar Grippotyphosa LipL32 polyclonal antiserum. As expected, a protein of ∼32 kDa was seen in *L. interrogans* serovar Icterohaemorrhagiae strain HAI188 (Figure 4). Surprisingly, a single, well-defined protein of ∼27 kDa, less than the expected molecular mass of this protein, was detected in *“L. licerasiae”* strain VAR 010^T^. Western blot analysis showed a 32 kDa band that co-migrated with the *L. interrogans* LipL32 in *L. broomii*, *L. weilii*, and *L. fainei* but failed to demonstrate any band in *L. wolbachii*, *L. biflexa*, and *Turneriella parva* (data not shown but provided for review). The unique size of the LipL32-cross reactive protein in *“L. licerasiae”* serovar Varillal strain VAR 010^T^ supports lack of contamination of this culture by another LipL32-containing leptospire. To further rule out the possibility that the cultures may have been contaminated by a known pathogenic *Leptospira* known to express *LipL32*, serological typing and 16S rRNA gene sequencing were repeated on all cultures, which confirmed their expected identities (data not shown).

### Experimental animal infections

**Figure 5:**
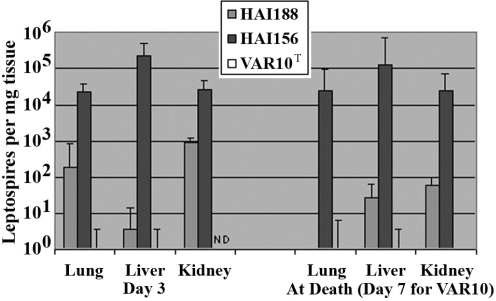
Real time quantitative PCR analysis of experimental leptospiral infections of hamsters. HAI188 and HAI156, strains *L. interrogans* serogroups Icterohaemorrhagiae and Canicola isolated from patients in Iquitos, Peru. VAR10, *“L. licerasiae”* serovar Varillal strain VAR 010^T^. HAI188 and HAI156 caused a severe moribund state at days 4–5; none of the animals with VAR 010^T^ exhibited any signs of illness. Three 25 mg samples of each tissue were analyzed and error bars indicate the standard deviations of these three samples per tissue.

Both *L. interrogans* serovar Canicola strain HAI156 and *L. interrogans* serovar Icterohaemorrhagiae strain HAI188 caused severe disease in hamsters infected intraperitoneally with 10^8^ leptospires. HAI156- and HAI188- infected hamsters were sick on day 3 following challenge and moribund by day 5. In contrast, hamsters infected with *“L. licerasiae”* serovar Varillal strain VAR 010^T^ showed no sign of illness (data not shown). Quantitative real time PCR detected high levels of leptospires in organs of hamsters infected with HAI156- and HAI188, but leptospires were nearly completely eliminated by day 3 after infection in liver, lungs and kidneys of hamsters infected with *“L. licerasiae”* serovar Varillal strain VAR 010^T^ (Figure 5), showing a major difference in virulence between these leptospiral species. A lack of symptomatic infection was found with experimental infection of more than 50 additional hamsters, as well as guinea pigs and SCID mice, with *“L. licerasiae”* serovar Varillal strain VAR 010^T^ (data not shown).

### Prevalence of *Leptospira licerasiae* serovar Varillal seropositivity in the Iquitos region

**Figure 6:**
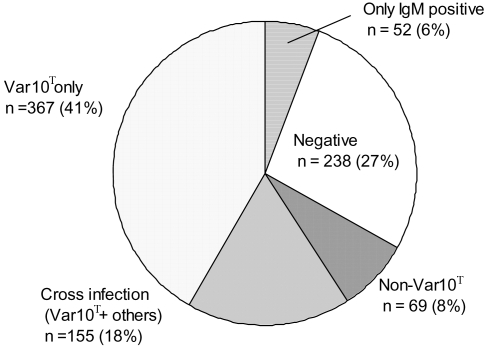
Seroprevalence of “*Leptospira licerasiae*” serovar Varillal in acute febrile patients in Iquitos, Peruvian Amazon (n = 881). Var 10 = *“Leptospira licerasiae”* serovar Varillal strain VAR 010^T^. Criteria for serological diagnosis of acute *Leptospira* infection: 1) IgM positive by ELISA in either acute or convalescent sera; 2) Conversion in the microscopic agglutination test (MAT) from negative to positive (1∶100 or greater); 3) Single MAT titer of 1:800 or greater; or 4) Four-fold rise in MAT titer.

During the study period, 1831 consecutive febrile patients were enrolled. Within these 1831 febrile patients on the data (means, including those with >2 weeks of febrile illness and one sample only), 881 had a second serum sample available between 10 and 70 days after the first sample. Of these, 516 (58.6%) met criteria for acute leptospirosis. Of these, 367 (41%) reacted to “*L. licerasiae*” serovar Varillal strain VAR 010^T^ only or had mixed reactions with “*L. licerasiae*” serovar Varillal strain VAR 010^T^ and other serovars (155, 18%) with diagnostic titers highest against “*L. licerasiae*” serovar Varillal strain VAR 010^T^ (Figure 6). The median percentage of febrile patients seropositive for *“L. licerasiae”* serovar Varillal strain VAR 010^T^ was 29% and the interquartile range was 23–36%. A single high MAT titer against “*L. licerasiae*” serovar Varillal strain VAR 010^T^ (≥1∶800) was found in 40 patients in the acute sample, 57 in the second sample, and 16 patients had a titer of ≥1∶800 in both.

Apart from the high rate of “*L. licerasiae*” serovar Varillal seroreactivity in the acute febrile population in Iquitos, we found serological evidence of seroreactivity in sera from 11 distinct geographic locations in Peru (Table 3). Though seroreactivity was not as common (22/344; 6.7% of seropositives) as in Iquitos, this finding does illustrate the widespread distribution of seroreactivity in Peru.

**Table 3:** *“Leptospira licerasiae”* serovar Varillal Seroreactivity in Acute Leptospirosis Patients from Different Regions of Peru.

Region of Peru	Number of Febrile Patients Studied	Number Diagnosed with Acute Leptospirosis	Number Seropositive against *“L. licerasiae”* Serovar Varillal 10
Cajamarca-Jaen	180	57	0
Amazonas-Bagua	262	103	0
Huanuco	15	2	0
Piura	16	5	0
Huanuco-Tingo Maria	32	4	0
Cusco	10	5	0
San Martin	21	9	1
Ayacucho-San Francisco	162	97	15
Junin	7	5	0
Ucayali	128	40	6
Lima Norte	41	17	1
**Totals**	874	344	23

### Specificity of MAT for anti-*Leptospira licerasiae* serovar Varillal antibodies

Due to the high frequency and titer of antibodies to “*L. licerasiae*” serovar Varillal strain VAR 010^T^, there was concern about the possibility that this leptospire might be cross-reactive with other organisms or that humans might have natural antibodies to this leptospire, so that seropositivity would be spurious and falsely positive. Fifty randomly collected, de-identified sera collected from inpatients at UCSD Medical Center were tested for antibodies against “*L. licerasiae*” serovar Varillal strain VAR 010^T^. There was no agglutination observed. We tested 180 sera collected from a serosurvey of healthy subjects in the north Lima town of Puente Piedra; of these, 2 had titers of 1∶50, the rest being negative.

## Discussion

Here we report isolation of a new species of *Leptospira* with novel biological characteristics that caused in humans a non-specific syndrome of undifferentiated fever. We showed definitively through serological and molecular analysis using 16S rRNA gene sequencing and pulsed field gel electrophoresis that this new leptospire, provisionally named “*Leptospira licerasiae*” serovar Varillal strain VAR 010^T^, is antigenically unique, is a significant cause of acute leptospirosis in the Peruvian Amazon region of Iquitos, and has a *Rattus* reservoir. Recognition of “*Leptospira licerasiae”* serovar Varillal strain VAR 010^T^ as a new serovar is supported by the lack of agglutination of this strain by any serogroup reference serum and the lack of reactivity of anti- VAR 010^T^ serum raised in rabbits against the serovars of *Leptospira* strains representing the nearly comprehensive and standard panel of leptospiral serogroups. A similar situation was found with *L. fainei* serovar Hurstbridge, where the following evidence was adduced in support of this novel serovar: lack of significant cross-agglutination was observed with reference antisera representing the 24 pathogenic serogroups and the main saprophytic ones; lack of agglutination by antiserum raised against one of the strains against any serogroup [19]. The serological characterization of the new serovar, Varillal, was conducted in two laboratories, one of which was the WHO/FAO/OIE Collaborating Centre for Reference and Research on Leptospirosis, Australia and Western Pacific Region, fulfilling the requirements for recognition of new serovars by the International Committee on Systematics of Prokaryotes, Subcommittee on the Taxonomy of *Leptospiraceae* [28].

The genus *Leptospira* presently consists of 13 named species and 4 unnamed genomospecies [19],[25],[29],[30]. Phylogenetic analysis reveals three clades, representing species that contain pathogenic serovars, non-pathogenic serovars and an intermediate group [19]. The latter clade comprises three species, *Leptospira broomii*, *Leptospira inadai* and *Leptospira fainei* [19],[26],[30]. Based on phylogenetic analysis, *L. licerasiae* is classified as an intermediate leptospiral species. Nonetheless, “*L. licerasiae”* serovar Varillal strain VAR 010^T^ shares properties with pathogenic *Leptospira* such as sensitivity to 8-azaguanine, has a LipL32-related protein as revealed by Western and Southern blots, but does not appear to contain a *LigA*-related gene as determined by Southern blot. In contrast to *L. interrogans, “L. licerasiae”* serovar Varillal strain VAR 010^T^ grew rapidly (similar to *L. biflexa*), did not grow significantly *in vivo*, and did not cause observable disease in experimentally infected animal. These observations suggest important biological and virulence differences between pathogenic and intermediate *Leptospira.*

Leptospirosis is typically thought of as an occupational disease originating from contact with water, soil or vegetation contaminated with the infected urine of carrier animals. The literature in recent years has shown that in under-developed areas of the world it is associated with environmental exposure during activities of daily living.^1,22^ Neither patient had an occupation that would be considered a risk factor for leptospirosis. Patient A in this study was a food vendor and had contact with obvious risk factors having frequented a market area (Belen) with poor sanitation and a high density of rats, and bathed in a natural pool, as there is no running water in her village. Patient B was a female student/domestic worker who lived in the city, did not frequent Belen (the urban slum area of Iquitos) and did not engage in other behavior that would place her at particular risk for contracting leptospirosis. However, both patients recalled seeing rats in and around their homes. Patient B did raise dogs and chickens, and the dogs urinated within the house. There were no established social or professional links between the patients, and their infections occurred in different places and times. Both cases presented with a mild, self-resolving febrile illness without secondary complications and show the ubiquity of exposure as part of the activities of daily living in this region.

Both patients initially had negative MAT and IgM results in their acute serum sample. While MAT of convalescent serum from Patient B was initially positive to a variety of serogroups, both acute and convalescent sera from Patient A were negative. However, when the test was repeated with the patient's own isolate, Patient A was found to have circulating leptospiral antibodies. This pattern of leptospiral seroreactivity, known to be a common problem in the diagnosis of leptospirosis,^2^ underscores the importance of including region-specific leptospiral isolates in the panel of strains used in MAT for diagnosing leptospirosis and determining its true burden.

A curious finding in this study is that isolation of “*L. licerasiae*” serovar Varillal from humans was rare, only being obtained from 2 of 881 febrile patients, despite the far higher seroprevalence rate of antibodies to this serovar. Some might raise the concern that this rare isolation rate could reflect a laboratory contamination with this leptospiral species. We believe this possibility is unlikely for two reasons. First, the patients from whom these isolates were obtained seroconverted to this novel serovar: patient 1 seroconverted to *L. licerasiae* serovar VAR 010^T^ but to no other leptospiral antigen by MAT, while patient 2 seroconverted with the highest titer against her own isolate of *L. licerasiae* at a titer higher than other leptospires. Second, we never obtained an isolate of *L. licerasiae* in any other culture of human or animal specimens other than those reported here, making the possibility of contaminated culture medium negligible. While the biological basis for the rare isolation of *L. licerasiae* remains speculative, we propose two hypotheses. First, it is possible that the two patients from whom this leptospire was isolated had an undefined, undetermined genetic predisposition that led to higher leptospiremia or failure to clear this relatively less virulent leptospire after exposure. Second, it is possible that varying degrees of heterologous, cross-reacting, anti-leptospiral immunity exist in the study population. This latter hypothesis is supported by the very high prevalence of anti-leptospiral antibodies in the Iquitos region, likely due to ubiquity of leptospiral exposure. It may be that these two patients, for some reason, never had been exposed to *Leptospira*, and thus were immunologically naïve and thus predisposed to a higher level of leptospiral bacteremia. Further prospective, population-based studies are needed to address these important questions.

This prospective study of acute febrile illness in Peru has shown that *“L. licerasiae”* is an important cause of fever in the Iquitos area and its surroundings, as evidenced by the number of patient sera that reacted predominantly or solely with serovar Varillal (298/425; 70%). Isolation of *“L. licerasiae”* from rats suggests that this leptospiral species has at least *Rattus* spp. as a major reservoir host; we have not found this leptospiral species in other rodent, bat and marsupial species in the Peruvian Amazon (Dr. Monica Diaz and J.M. Vinetz et al, data not shown). Domestic rats are common in Iquitos: *R. norvegicus* and *R. rattus* are closely associated with human settlements in the area. *R. norvegicus* is more often encountered in urbanized areas, while *R. rattus* is the predominant rural species (data not shown). Six isolates identical to those isolated from both patients were recovered from rats caught in Belen, a city slum where sanitation is poor, rats are common and the risk of transmission to man is high. A further two VAR 010^T^-related isolates were recovered from rural rats. The isolation of a strain common to rats and found in 2 clinical cases, the ubiquity of the rat in Iquitos and the poor sanitation in most areas make the rat the likely source of leptospires in Iquitos. In other studies, we have succeeded in isolating *L. interrogans* serovar Icterohaemorrhagiae from 22 to 48% of peri-domestic *Rattus* species in villages near to and within urban areas of Iquitos (unpublished observations).

Molecular and serological analysis of human- and rat-derived strains revealed that they comprise a single novel leptospiral species and serovar. The fact that the PFGE fingerprint patterns were found to be identical to each other, but did not match any of the patterns in the CDC database (P.N. Levett and R. Galloway, unpublished data) supports our contention that the strains are novel leptospires. The isolates are members of a new serovar and serogroup as none were agglutinated by any of the reference anti-sera in our panel, although they had trace reactions to serogroup Hurstbridge. Given the lack of reactivity to the leptospiral serogroups represented by the rabbit reference sera used in the present study, the reference serological test, the cross-absorption agglutination test, was not necessary to define Varillal as a new serovar or antigenic type, similar to what was found for *L. fainei* serovar Hurstbridge [19]. Phylogenetic analysis of 16S rRNA gene sequence demonstrated that the strains comprised a homogenous genetic group separate from all other described leptospiral species. These strains, much like the recently described *L. broomii* [30], *L. fainei* serovar Hurstbridge [19] and *L. inadai* serovar Lyme [31], are intermediate between the two larger saprophytic and pathogenic groups of *Leptospira* and, as such, share characteristics similar to both pathogenic and saprophytic leptospires. DNA-DNA hybridization further confirmed that *L. licerasiae* is a new *Leptospira* species. Our logic was similar to that taken in using DNA-DNA hybridization to further confirm *L. broomii* as a new *Leptospira* species [30]. Because 16S rRNA gene sequencing places *L. licerasiae* into the intermediate *Leptospira* group close to *L. fainei, L. broomii*, etc., the only relevant DNA-DNA hybridization analysis is to differentiate the closest known clade partners identified by 16S rRNA gene sequence, so as to be able to confirm the distinctness of these 16S-rRNA gene-defined intermediate *Leptospira* species. The DNA-DNA hybridization analysis reported here does indeed confirm the distinctness of *L. licerasiae* from the other known intermediate *Leptospira*.

We report the existence of a new species of leptospire, provisionally named “*Leptospira licerasiae”* serovar Varillal, which causes acute leptospirosis in the Peruvian Amazon. We have proposed this name to recognize the contribution of Professor Julia Liceras de Hidalgo who obtained the first leptospiral isolates in Peru [7], [32]–[37]. We propose a new serogroup, Iquitos, based on the lack of agglutination with a comprehensive panel of reference antisera comprised of all serogroups except for Lyme and Sehgali (which in the case of serovar Lyme had cross-reaction with serovar Celledoni at a titer of 1∶400 [31], and in the case of Sehgali had a broad level of cross-reactivity 25 serovars and 12 serogroups ranging from titers of 1∶80 to 1∶1280 [38]).

Based on serological data that take advantage of its antigenic uniqueness, “*Leptospira licerasiae”* serovar Varillal appears to be an important cause of leptospirosis in the Peruvian Amazon region, but is uncommon elsewhere in Peru. The peridomestic rat is likely the major reservoir of this new species. Elucidation of virulence differences between pathogenic and intermediate leptospires will provide insight into leptospiral evolution and disease mechanisms, and may contribute to the control and amelioration of leptospirosis in the developing world.

### Note on Taxonomy

To fulfill the rules of the International Code of Nomenclature of Bacteria [Lapage SP, Sneath PHA, Lessel EF, Skerman VBD, Seeliger HPR, Clark WA. International code of nomenclature of bacteria (1990 revision). Washington, DC: American Society for Microbiology, 1992.], we provide the following description of the novel species identified in this report.

**Description of** ***Leptospira licerasiae*** **sp. nov.** *Leptospira licerasiae* (li.ce.ra' si.ae. N.L. fem gen. n. licerasiae of Liceras, to honor Dr. Julia Liceras de Hidalgo, who obtained the first leptospiral isolates in Peru). Isolated from the blood of human patients with febrile illness and from kidneys of rats in Peru. Morphology is as described previously for the genus [25],[39]. The G+C ratio is 43.9 mol%. The type strain is VAR 010^T^ ( = ATCC BAA-1110^T^ = WPR VAR 010^T^), and has been deposited at the American Type Culture Collection, Manassas, Virginia, the National Veterinary Services Laboratory, U.S. Department of Agriculture, Ames, Iowa, and the WHO/FAO/OIE Collaborating Centre for Reference and Research on Leptospirosis, Australia and Western Pacific Region.

### Database deposition

The 16S rRNA sequences for the *Leptospira licerasiae* isolates reported in this paper have been deposited in GenBank with the following accession numbers (strain, accession number): CEH006, EF612278; CEH011, EF612279; CEH033,EF612280; CEH044, EF612281; CEH162, EF612282; MMD735, EF612283; VAR 010^T^, EF612284; CEH010, EF612285; CEH174, EF612286; MMD835, EF612287; HAI029, EF612288.

## Supporting Information

Alternative Lanuage Abstract S1 Translation of the Abstract into Spanish by Jessica N. Ricaldi
(0.03 MB DOC)

Figure S1 Schematic of Leptospiral 16rDNA Gene Sequencing Strategy
(9.45 MB TIF)

Figure S2 Assembly of One of 10 Identical 16S rDNA Sequences of *L. licerasiae*, Strain CEH033
(0.06 MB PDF)

Table S1 Results of Serogroup Screening Against VAR 010^T^ as Determined by the WHO/FAO/OIE Collaborating Centre For Reference & Research on Leptospirosis, Brisbane, Australia
(0.05 MB DOC)
