# Supplementary material for: Human Leptospirosis Caused by a New, Antigenically Unique Leptospira Associated with a Rattus Species Reservoir in the Peruvian Amazon
Source: PLoS Negl Trop Dis. 2008 Apr 2;2(4):e213. doi: 10.1371/journal.pntd.0000213 (PMC2271056; doi:10.1371/journal.pntd.0000213)
Supplement: Alternative Lanuage Abstract S1 — Translation of the Abstract into Spanish by Jessica N. Ricaldi (0.03 MB DOC) [file pntd.0000213.s001.doc]

**Resumen**

Como parte de un estudio prospectivo sobre leptospirosis y biodiversidad de *Leptospira* en la Amazonia peruana, se aisló una especie nueva de *Leptospira* a partir de pacientes con enfermedad febril aguda. También se identificó esta especie en ratas peridomesticas (*Rattus* *norvegicus,* 6 aislamientos*,* *R. rattus*, 2 aislamientos) obtenidas en áreas urbanas, peri urbanas y rurales de la región alrededor de Iquitos. Se determinó que esta especie es nueva utilizando tipificación serológica, secuenciamiento del gen ribosomal 16S, electroforesis de campo pulsado, y análisis de hibridación DNA-DNA. Hemos llamado a esta especie “*Leptospira licerasiae”* serovar Varillal, y hemos determinado que esta filogenéticamente relacionada con, pero es genéticamente distinta a otras leptospiras intermedias como *L. fainei* y *L. inadai*. La cepa tipo es serovar Varillal cepa VAR 010 ^T^, la cual se ha depositado en colecciones de cultivos internacionalmente asequibles. Usando la prueba de micro aglutinación (MAT) se determinó que “*Leptospira licerasiae”* serovar Varillal es antigénicamente distinta de todos los serogrupos conocidos de *Leptospira,* con excepción de reacción cruzada a niveles bajos (títulos de 1:100) con antisuero de conejo anti-*L.fainei*  serovar Hurstbridge. No se detectó *LipL32* en “*Leptospira licerasiae”* serovar Varillal utilizando Reacción en Cadena de Polimerasa (PCR), pero si se detectó utilizando Southern y Western blot. En el Western blot, la proteína detectada fue significativamente mas pequeña (27 kDa) que la de *L. interrogans* y *L. kirschneri* (32 kDa). El aislamiento en humanos fue infrecuente (2/45 aislamientos de *Leptospira* a partir de 881 muestras de pacientes febriles ), pero fue común (30%) encontrar títulos altos de anticuerpos en MAT contra *“L. licerasiae”* serovar Varillal en pacientes que cumplían criterios serológicos para el diagnóstico de leptospirosis aguda en la región de Iquitos, y poco frecuente (7%) en otras regiones del Perú. Esta nueva especie de *Leptospira* refleja la biodiversidad que existe en la Amazonia y ha evolucionado para convertirse en una causa importante de leptospirosis en la Amazonia peruana.
