# Supplementary figures and images for: Human Leptospirosis Caused by a New, Antigenically Unique Leptospira Associated with a Rattus Species Reservoir in the Peruvian Amazon
Source: PLoS Negl Trop Dis. 2008 Apr 2;2(4):e213. doi: 10.1371/journal.pntd.0000213 (PMC2271056; doi:10.1371/journal.pntd.0000213)

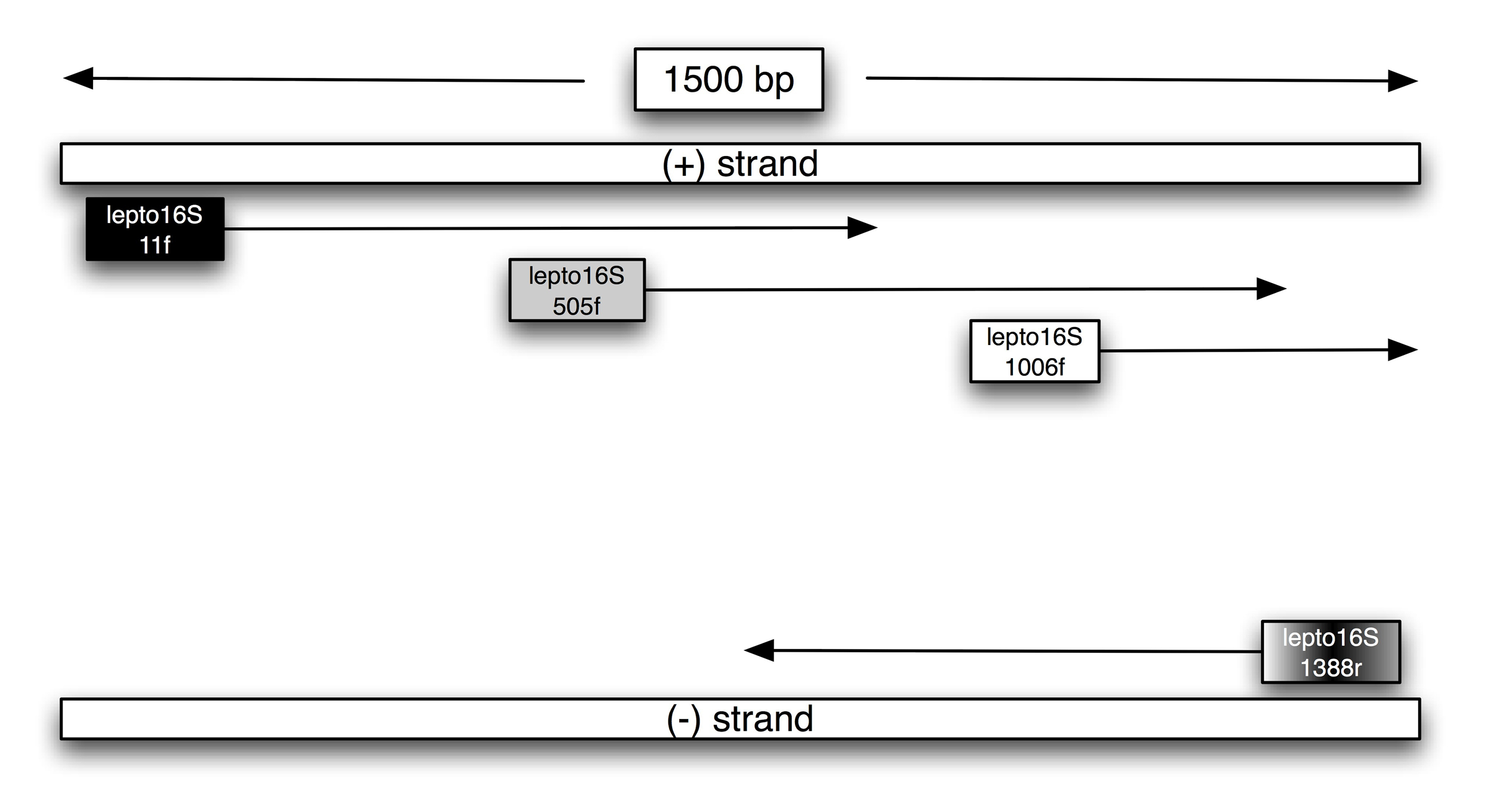

Supplement: Figure S1 — Schematic of Leptospiral 16rDNA Gene Sequencing Strategy (9.45 MB TIF) [file pntd.0000213.s002.tif]

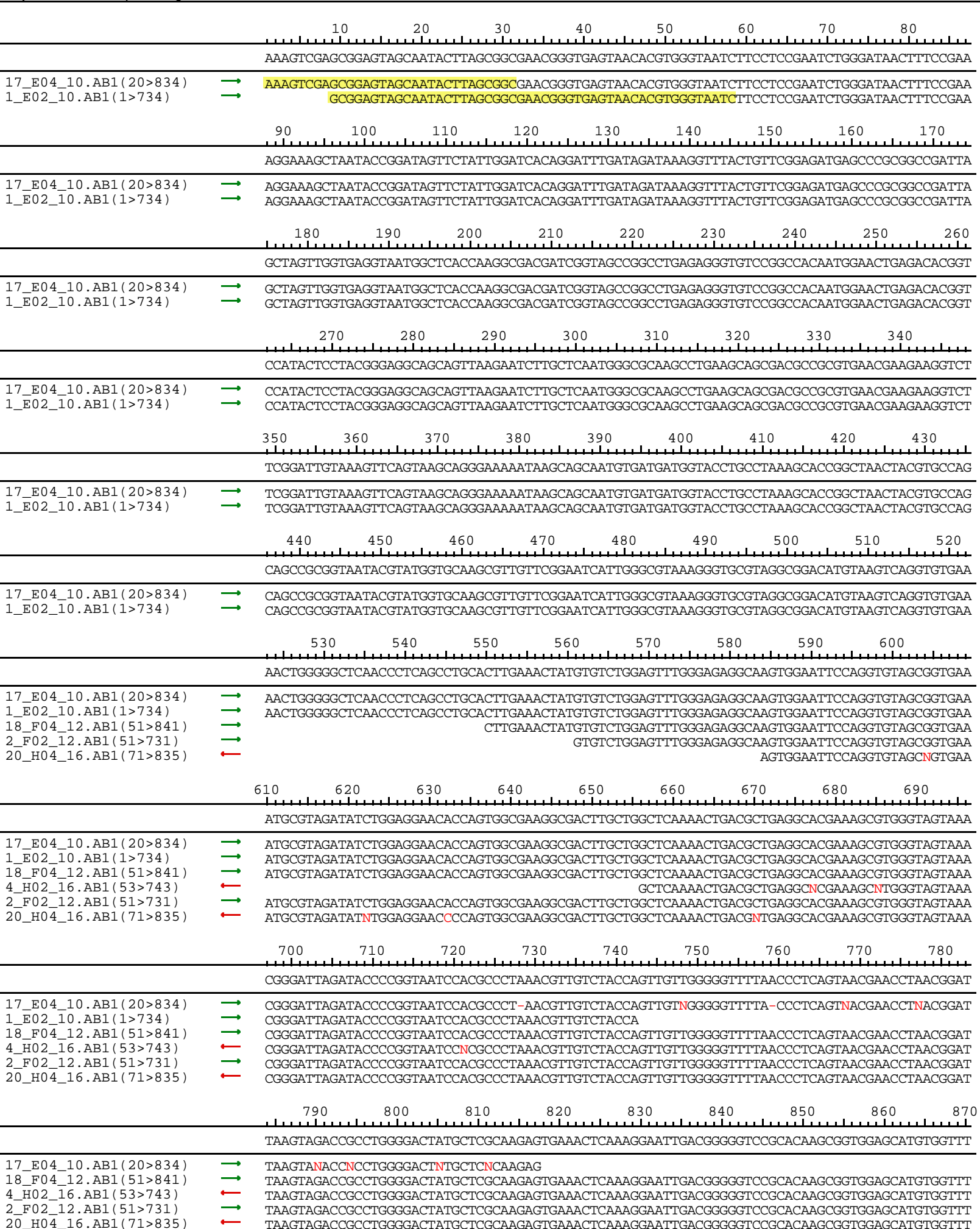

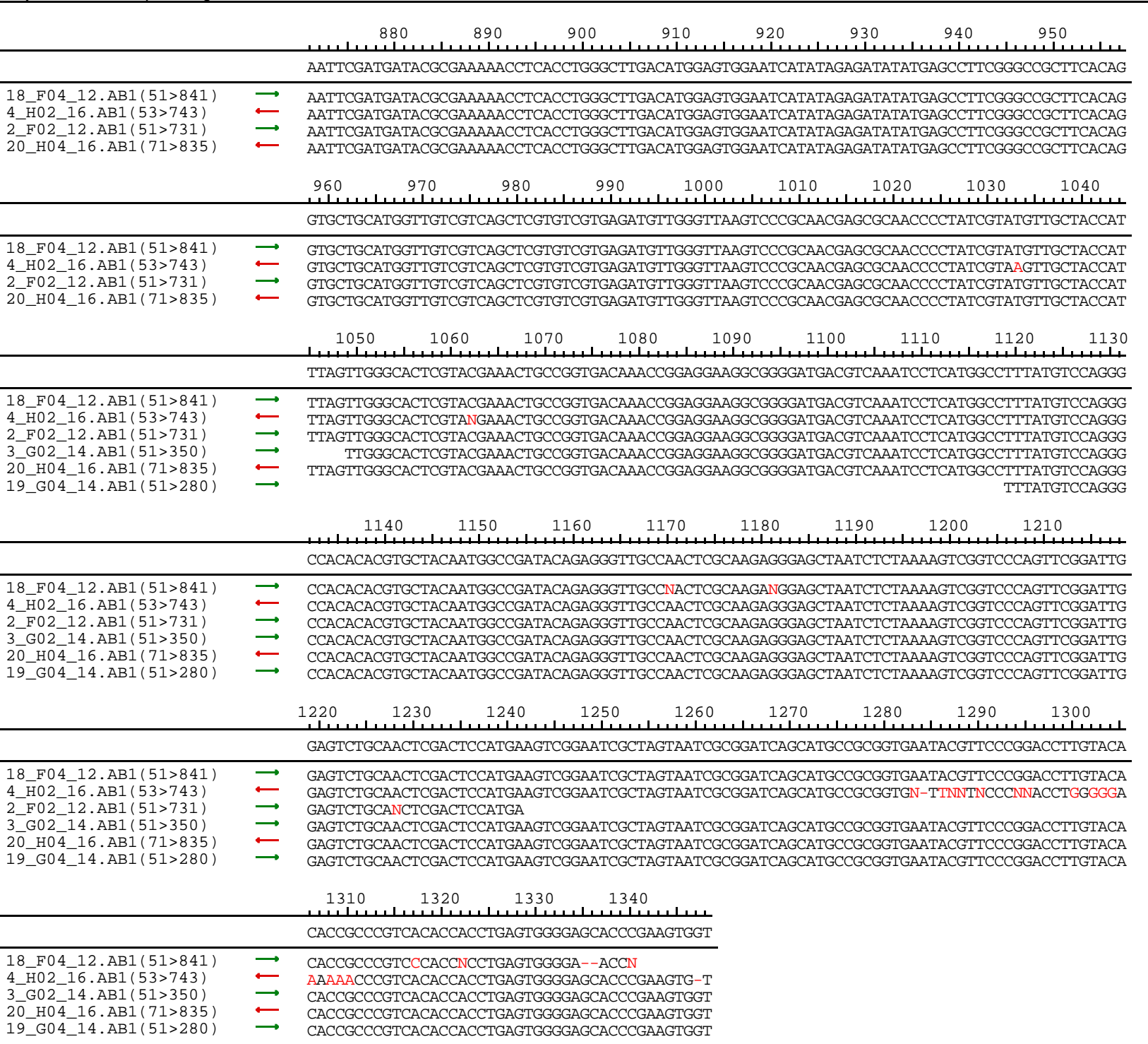

Supplement: Figure S2 — Assembly of One of 10 Identical 16S rDNA Sequences of L. licerasiae, Strain CEH033 (0.06 MB PDF) [file pntd.0000213.s003.pdf]
