# Supplementary material for: Human Leptospirosis Caused by a New, Antigenically Unique Leptospira Associated with a Rattus Species Reservoir in the Peruvian Amazon
Source: PLoS Negl Trop Dis. 2008 Apr 2;2(4):e213. doi: 10.1371/journal.pntd.0000213 (PMC2271056; doi:10.1371/journal.pntd.0000213)
Supplement: Table S1 — Results of Serogroup Screening Against VAR 010T as Determined by the WHO/FAO/OIE Collaborating Centre For Reference & Research on Leptospirosis, Brisbane, Australia (0.05 MB DOC) [file pntd.0000213.s004.doc]

**Supplementary Table 1S. Results of Serogroup Screening Against VAR 010^T^ as Determined by the WHO/FAO/OIE Collaborating Centre For Reference & Research on Leptospirosis, Brisbane, Australia**

| **Species** | **Serovar** | **Reference Strain** | **Serogroup** | **Titers against VAR 010^T^** |
| --- | --- | --- | --- | --- |
| *L. interrogans* | Copenhageni | M20 | Icterohaemorrhagiae | <50 |
| *L. borgpetersenii* | Javanica | Veldrat Batavia 46 | Javanica | <50 |
| *L. weilii* | Celledoni | Celledoni | Celledoni | <50 |
| *L. interrogans* | Canicola | Hond Utrecht IV | Canicola | <50 |
| *L. borgpetersenii* | Arborea | Arborea | Ballum | <50 |
| *L. interrogans* | Zanoni | Zanoni | Pyrogenes | <50 |
| *L. interrogans* | Robinsoni | Robinson | Pyrogenes | <50 |
| *L. kirschneri* | Cynopteri | 3522C | Cynopteri | <50 |
| *L. interrogans* | Bulgarica | Nikolaevo | Autumnalis | <50 |
| *L. interrogans* | Djasiman | Djasiman | Djasiman | <50 |
| *L. interrogans* | Australis | Ballico | Australis | <50 |
| *L. interrogans* | Pomona | Pomona | Pomona | <50 |
| *L. kirschneri* | Grippothyposa | Moskva V | Grippotyphosa | <50 |
| *L. interrogans* | Krematos | Kremastos | Hebdomadis | <50 |
| *L. interrogans* | Szwaijak | Szwajizak | Mini | <50 |
| *L. interrogans* | Hardjo | Hardoprajitno | Sejroe | <50 |
| *L. interrogans* | Medanensis | Hond HC | Sejroe | <50 |
| *L. interrogans* | Bataviae | Swart | Bataviae | <50 |
| *L. borgpetersenii* | Tarrasovi | Perepelitsin | Tarassovi | <50 |
| *L. noguchii* | Panama | CZ 214 | Panama | <50 |
| *L. santarosai* | Shermani | 1342K | Shermani | <50 |
| *L. biflexa* | Patoc | Patoc I | Semaranga | <50 |
| *L. broomi* |  | Feral |  | <50 |
| *L.meyeri* | Ranarum | ICF | Ranarum | <50 |
| *L.noguchi* | Orleans | LSU 2580 | Lousisana | <50 |
| *L.weilii* | Sarmim | Sarmin | Sarmin | <50 |
|  | Manhao 4 | Li 130 | Manhao | <50 |
